# Clinical Features and Associated Factors of Lupus Myocarditis: A Case‐Control Study

**DOI:** 10.1002/iid3.70436

**Published:** 2026-04-13

**Authors:** Dong Yan, Siping Li, Mengxue Yan, Zhichun Liu, Leixi Xue

**Affiliations:** ^1^ Department of Rheumatology and Immunology Second Affiliated Hospital of Soochow University Suzhou China

**Keywords:** echocardiography, myocarditis, rituximab, Systemic lupus erythematosus

## Abstract

**Objectives:**

This study aimed to explore the clinical characteristics of patients with lupus myocarditis (LM) and to evaluate the efficacy of rituximab (RTX) in LM treatment.

**Methods:**

The medical records of all patients with LM admitted to our hospital between January 2012 and March 2025 were retrospectively analyzed. Two control groups were established by randomly matching patients by sex and age at a 1:1 ratio: patients with systemic lupus erythematosus (SLE) without LM and patients with non‐SLE myocarditis. The SLE disease activity index 2 K (SLEDAI 2 K) score and Systemic Lupus Erythematosus Disease Activity Score (SLE‐DAS) were calculated to evaluate SLE disease activity.

**Results:**

A total of 22 patients with LM were enrolled. Patients with LM had a higher incidence of lupus nephritis and a higher positivity rate for anti‐SSB antibodies than those with SLE without LM. Furthermore, patients with SLE with LM had significantly higher SLE‐DAS than those without LM, although no statistical difference in SLEDAI 2 K score was observed between the groups. Wall motion abnormalities, valvular regurgitation, and decreased left ventricular ejection fraction (LVEF) were more frequent in patients with LM than in those with non‐SLE myocarditis. All patients with LM received corticosteroid treatment, with three of them receiving RTX in addition to standard therapy. With a median follow‐up of 4 (range, 1–24) months, 2 patients (9.1%) died due to heart failure, and the remaining 20 achieved symptom remission. Moreover, 13 patients underwent follow‐up echocardiography, which showed a significant improvement in LVEF. The three patients treated with RTX achieved clinical improvement within a mean of 2 weeks, enabling rapid glucocorticoid tapering.

**Conclusions:**

Patients with LM more frequently present with lupus nephritis and positive anti‐SSB antibodies, are more likely to have echocardiographic abnormalities, and exhibit a higher mortality rate. In addition, RTX is a promising drug for LM treatment.

## 1 Introduction

Systemic lupus erythematosus (SLE) is a systemic autoimmune disease marked by excessive immune system activation and causes damage to various tissues and organs, including the dermatological, renal, neuropsychiatric, hematological, and cardiovascular systems [1]. In SLE, all cardiac structures are affected, with pericarditis being the most prevalent cardiac manifestation [2]. Lupus myocarditis (LM) is a more severe clinical condition than pericarditis owing to its potential impact on cardiac function, with a 17.3% mortality rate [3]. Approximately 9% of patients with SLE present with LM. However, postmortem studies have demonstrated subclinical myocardial involvement in up to 57% of patients with SLE [4].

Despite the substantial clinical importance of LM, the underlying mechanisms remain poorly understood. Autoantibodies are considered to play a pivotal role in LM, similar to their contributions to damage in other organs and tissues in SLE. Autoantibodies targeting heart‐specific antigens, such as cardiac myosin heavy chain (MyHC), are also potentially associated with myocardial injury in SLE. When the PD‐1/PD‐L1 pathway was genetically blocked, MRL and MRL/lpr mice, which are well‐established SLE mouse models, spontaneously developed autoimmune myocarditis, accompanied by the production of a high titer of anti‐MyHC antibodies [5, 6]. As regards cellular signaling pathways, the mammalian target of rapamycin (mTOR) signaling may be involved in the pathological process of LM [7]. In experimental autoimmune myocarditis, rapamycin specifically targets pathogenic Cxcl9⁺ macrophages by disrupting the mTORC1–C/EBPβ axis and limiting their differentiation from Plac8^+^ monocytes, thereby preserving cardiac function and reducing myocardial inflammation and fibrosis. Concurrently, it protects cardiomyocytes by blocking C/EBPβ‐dependent OSM‐mediated macrophage–cardiomyocyte crosstalk. A clinical study reported that patients with SLE with myocardial injury had higher levels of serum interleukin (IL)−1 receptor antagonist, IL‐17, IL‐18, and soluble vascular cell adhesion molecule‐1 than those with SLE without myocardial injury; however, the role of these factors in the pathological progression of LM remains unclear [8].

The gold standard tool for diagnosing LM is endocardial biopsy (EMB), as it enables the distinction of LM from other causes of myocarditis [9]. However, biopsy results are sometimes nonspecific and may resemble other types of immune‐mediated myocarditis, reducing the clinical value of EMB. Moreover, the invasive nature of EMB and associated procedural risks limit its use in routine clinical practice. To address this issue, noninvasive diagnostic methods, such as cardiac imaging and the use of serological immune markers and circulating biomarkers, are now being used, clinically tested, or currently developed [10].

Glucocorticoids (GCs) and immunosuppressants are frequently used in LM therapy. However, there is currently no international consensus on its management. Furthermore, there is limited evidence for LM treatment with biological agents. The distinctive features of LM are less well understood than those of myocarditis secondary to other diseases. Therefore, this study aimed to describe the clinical features and outcomes of LM to obtain potential new information on LM and to evaluate the efficacy of rituximab (RTX) in LM.

## 2 Materials and Methods

### 2.1 Patients

This study retrospectively enrolled patients with SLE with LM who were admitted to the Second Affiliated Hospital of Soochow University between January 2012 and March 2025. To identify risk factors and potentially novel LM characteristics, two control groups were established by randomly matching patients by sex and age at a 1:1 ratio: patients with SLE without LM and patients with non‐SLE myocarditis.

All patients with SLE met at least one of the following classification criteria: the American College of Rheumatology (ACR) 1997 revised criteria, 2012 Systemic Lupus International Collaborating Clinics classification criteria, or 2019 European League Against Rheumatism/ACR classification criteria [11]. The diagnosis of LM required fulfillment of two of the following criteria: (a) elevated serum troponin levels, (b) new‐onset systolic dysfunction and wall motion abnormalities in echocardiography, and (c) evidence of acute edema or delayed enhancement in magnetic resonance imaging. The exclusion criteria for LM were as follows: (a) congenital heart disease, such as cardiomyopathy, congenital heart disease, or cardiac insufficiency; (b) coronary artery disease; (c) cardiac surgery history; (d) presence of other secondary factors, such as viral infection, longstanding hypertension, or toxin exposure; (e) moderate‐to‐severe valvular disease; and (f) incomplete or missing data in medical records.

The study was approved by the Human Ethics Review Committee of the Second Affiliated Hospital of Soochow University and was conducted in accordance with the principles of the Declaration of Helsinki (JD–HG–2025–042). As the study was based on a review of medical records, the requirement for written informed consent was waived. Prior to analysis, patient data and records were de‐identified and anonymized.

### 2.2 Data Collection

Clinical data obtained from the medical records included those pertaining to demographics, organ involvement, laboratory tests, electrocardiograms (ECG), echocardiographic results, treatments, and clinical outcomes. The following laboratory parameters were tested: antinuclear antibody, anti‐dsDNA, anti‐Sm, anti‐U1RNP, anti‐Ro52, anti‐SSA, anti‐SSB, anti‐ribosomal P protein (rRNP), complement 3 (C3), C4, erythrocyte sedimentation rate (ESR), C‐reactive protein, troponin‐T, creatine kinase‐MB, N‐terminal pro‐B‐type natriuretic peptide (NT‐proBNP), and myoglobin. In addition, echocardiography data were acquired, including the left ventricular ejection fraction (LVEF), deceleration time of the early filling velocity, left ventricle end‐diastolic diameter, and tricuspid annular plane systolic excursion.

Furthermore, the SLE disease activity index 2 K (SLEDAI 2 K) score and the Systemic Lupus Erythematosus Disease Activity Score (SLE‐DAS) were calculated to evaluate the overall disease activity in SLE [12, 13], and the New York Heart Association (NYHA) classification was employed to ascertain clinical cardiac function.

### 2.3 Statistical Analysis

Statistical analysis was conducted using the SPSS software version 21.0 (IBM Inc., Armonk, USA). Quantitative variables with a normal distribution were expressed as mean ± standard deviation, whereas those with a non‐normal distribution were expressed as median and range. Continuous variables were analyzed using Student's *t*‐test or the Mann–Whitney *U* test, as appropriate. Categorical variables were expressed as frequencies and percentages, whereas intergroup differences were evaluated using the chi‐squared test or Fisher's exact test. A *p*‐value < 0.05 was considered statistically significant.

## 3 Results

### 3.1 Epidemiology

This study enrolled 22 patients with SLE and LM, of whom 20 (90.9%) were women. Their age at SLE onset was 42.3 ± 18.4, and the mean age at LM onset was 46.8 ± 22.3 years. The median duration of SLE was 2 (range, 0–40) years. LM was the initial manifestation in 11 (50.0%) patients with SLE (Table 1).

**Table 1:** Comparison of the clinical characteristics of patients with LM and patients with SLE without LM.

	LM (*n* = 22)	SLE without LM (*n* = 22)	*P*
Age, mean ± SD, years	50.1 ± 19.0	40.9 ± 14.8	0.080
Age at SLE onset, mean ± SD, years	42.3 ± 18.4	36.3 ± 15.6	0.248
Disease duration of SLE, Median (range)	2 (0–40)	1 (0–15)	0.791
Lupus clinical characteristics			
Nephropathy, *n* (%)	17 (77.3)	9 (40.9)	0.014
Mucocutaneous involvement, *n* (%)	11 (50)	12 (54.5)	0.673
Arthritis, *n* (%)	5 (22.7)	3 (13.6)	0.698
Neurological involvement, *n* (%)	0	1 (4.5)	0.312
Serositis, *n* (%)	3 (13.6)	0	0.233
Thrombocytopenia, *n* (%)	7 (31.8)	9 (40.9)	0.531
Hemolytic anemia, *n* (%)	1 (4.5)	0	1.000
Leukopenia, *n* (%)	3 (13.6)	8 (36.4)	0.082
Laboratory examination			
C3 (g/L), mean ± SD	0.50 ± 0.19	0.59 ± 0.24	0.218
C4 (g/L), mean ± SD	0.11 ± 0.06	0.12 ± 0.07	0.705
ESR (mm/H), mean ± SD	47.9 ± 30.4	25.9 ± 20.6	0.012
CRP (mg/L), Median (Range)	9.7 (0–132)	5.4 (1–25)	0.121
Anti‐dsDNA, *n* (%)	12 (54.5)	15 (68.2)	0.353
Anti‐Sm, *n* (%)	10 (45.5)	6 (27.3)	0.210
Anti‐U1RNP, *n* (%)	16 (72.7)	13 (59.1)	0.340
Anti‐rRNP, *n* (%)	8 (36.4)	3 (13.6)	0.082
Anti‐SSA, *n* (%)	16 (72.7)	10 (45.5)	0.066
Anti‐SSB, *n* (%)	8 (36.4)	2 (9.1)	0.031
Anti‐Ro52, *n* (%)	15 (68.2)	11(50)	0.220
Hydroxychloroquine intake, *n* (%)	12 (54.5)	15 (68.2)	0.353
Disease activity index			
SLEDAI‐2k, mean ± SD	13.41 ± 7.11	11.86 ± 4.98	0.408
SLE‐DAS, mean ± SD	29.86 ± 7.88	15.79 ± 9.66	< 0.001

Abbreviations: C, complement; CI, confidential interval; CRP, C‐reactive protein; ESR, erythrocyte sedimentation rate; LM, lupus myocarditis; OR, odds ratio; SLE, systemic lupus erythematosus; SLEDAI‐2k, Systemic Lupus Erythematosus Disease Activity Index‐2K; SLE‐DAS, Systemic Lupus Erythematosus Disease Activity Score.

### 3.2 Clinical Characteristics of Patients With LM

Table 2 presents the signs and symptoms of LM. The median time between SLE diagnosis and LM onset was 7.6 (range, 0–40) years. The most prevalent symptom of LM was chest tightness (63.6%), followed by weakness (59.1%), cough (50%), palpitation (31.8%), dyspnea (31.8%), and chest pain (4.5%). Overall, 10 patients (45.5%) were classified as NYHA Class III or IV.

**Table 2:** Comparison of the clinical characteristics of patients with LM and patients with non‐SLE myocarditis.

	LM (*n* = 22)	Non‐SLE myocarditis (*n* = 22)	*P*
Age at myocarditis onset, mean ± SD	46.8 ± 22.3	52.1 ± 16.4	0.381
Age at LM diagnosis, mean ± SD	47.8 ± 21.8	NA	
SLE duration at LM diagnosis, Median (range)	7.6 (0 – 40)	NA	
Symptoms			
Chest tightness, *n* (%)	14 (63.6)	18 (81.8)	0.176
Weakness, *n* (%)	13 (59.1)	10 (45.5)	0.365
Cough, *n* (%)	11 (50)	11 (50)	1.000
Palpitation, *n* (%)	7 (31.8)	8 (36.4)	0.750
Dyspnea, *n* (%)	7 (31.8)	10 (45.5)	0.353
Chest pain, *n* (%)	1 (4.5)	9 (40.9)	0.004
Main signs			
Peripheral edema, *n* (%)	7 (31.8)	5 (22.7)	0.498
Pulmonary rales, *n* (%)	7 (31.8)	7 (31.8)	1.000
NYHA classification			
I–II, *n* (%)	12 (54.5)	14 (63.6)	0.540
III–IV, *n* (%)	10 (45.5)	8 (36.4)	0.540
Laboratory examination			
CRP (mg/L), median (range)	11.1 (0–132.4)	7.8 (0.1–272.3)	0.830
Elevated troponin‐T, *n* (%)	14 (63.6)	17 (77.3)	0.680
Elevated NT‐proBNP, *n* (%)	16 (72.7)	14 (63.6)	0.465
Electrocardiogram			
Arrhythmia, *n* (%)	3 (13.6)	4 (18.2)	1.000
Sinus tachycardia, *n* (%)	3 (13.6)	7 (31.8)	0.150
ST‐segment abnormalities, *n* (%)	6 (27.3)	3 (13.6)	0.457
Echocardiography data			
LVEDD (mm), mean ± SD	47.74 ± 7.71	44.6 ± 5.09	0.313
Increased LVEDD (> 53 mm), *n* (%)	6 (27.3)	1 (4.5)	0.095
Left atrial enlargement, *n* (%)	8 (36.4)	9 (40.9)	1.000
Left ventricular enlargement, *n* (%)	6 (27.3)	1 (4.5)	0.095
Right atrial enlargement, *n* (%)	6 (27.3)	1 (4.5)	0.095
Right ventricular enlargement, *n* (%)	4 (18.2)	0	0.108
Valvular regurgitation, *n* (%)	8 (36.4)	2 (9.1)	0.031
Hydropericardium, *n* (%)	16 (72.7)	10 (45.5)	0.066
Tapse (mm), median (range)	19.2 (15–30)	19 (16–26)	0.305
Wall motion abnormalities, *n* (%)	16 (72.7)	5 (22.7)	0.001
LVEF < 55%, *n* (%)	8 (36.4)	2 (9.1)	0.031

Abbreviations: CRP, C‐reactive protein; LVEDD, left ventricle end‐diastolic diameter; LVEF, left ventricular ejection fraction; NT‐proBNP, N‐terminal pro‐B‐type natriuretic peptide; NYHA, New York Heart Association; Tapse, tricuspid annular plane.

Regarding biomarkers of myocardial injury, 14 (63.6%) patients exhibited elevated troponin‐T levels, with a median concentration of 39 (range, 3–1549) pg/mL. Elevated NT‐proBNP levels were detected in 16 (72.7%) patients.

None of the LM patients had undergone EMB. The ECG findings included arrhythmia and sinus tachycardia, each observed in three patients (13.6%). Furthermore, 6 (27.3%) patients showed nonspecific ST‐segment abnormalities.

Initial echocardiography was performed in all patients. Of them, 16 (72.7%) demonstrated hydropericardium, whereas 16 (72.7%) exhibited wall motion abnormalities (global or segmental hypokinesis). Moreover, 8 (36.4%) patients showed reduced LVEF (< 55%), and 8 (36.4%) exhibited valvular regurgitation (Table 2).

### 3.3 Comparison with Patients with SLE Without LM

The control group consisted of 22 patients with SLE without LM who were matched 1:1 by age and sex. Compared with the control group, the LM group had a significantly higher prevalence of lupus nephritis (LN) (77.3% vs. 40.9%, *p* = 0.014). In addition, the LM group showed higher ESR level (47.9 ± 30.4 vs. 25.9 ± 20.6 mm/h, *p* = 0.012) and higher positivity rate for anti‐SSB antibodies (36.4% vs. 9.1%, *p* = 0.031); the rate of anti‐SSA positivity in patients with LM was also higher but did not reach statistical significance (72.7% vs. 45.5%, *p* = 0.066) (Table 1).

Disease activity assessment using two different scales yielded varying results. The SLEDIA‐2K score revealed no statistical difference between the groups, whereas the SLE‐DAS was significantly higher in patients with LM than in those with SLE without LM (*p* < 0.001) (Table 1).

### 3.4 Comparison With Patients With Non‐SLE Myocarditis

The clinical features of patients with LM and those with non‐SLE myocarditis were compared. Exploratory comparative analyses between the groups revealed no significant differences in the NYHA classification, laboratory test results, and ECG findings. The incidence of chest pain was significantly lower in patients with LM than in those with non‐SLE myocarditis (4.5% vs. 40.9%, *p* = 0.004), whereas wall motion abnormalities (72.7% vs. 22.7%, *p* = 0.001) and reduced LVEF (36.4% vs. 9.1%, *p* = 0.031) were more common in patients with LM than in those with non‐SLE myocarditis. Moreover, patients with LM demonstrated a higher frequency of valvular regurgitation than those with non‐SLE myocarditis (36.4% vs 9.1%, *p* = 0.031) (Table 2).

### 3.5 Treatment and Outcome

All patients with LM were initially treated with high‐dose corticosteroids (as prednisone ≥ 1 mg/kg/d), and 4 (18.2%) of them received methylprednisolone pulse therapy. In addition, 3 patients were administered intravenous immunoglobulin (IVIG). As regards immunosuppressive therapy, mycophenolate mofetil (MMF) was administered to nine patients, cyclophosphamide (CTX) to eight, and leflunomide to two. Hydroxychloroquine (HCQ) was also administered to 12 patients. Furthermore, three patients were initially treated with RTX at a dose of 500 mg every fortnight, of whom two received two infusions and the other received three infusions.

**Figure 1:**
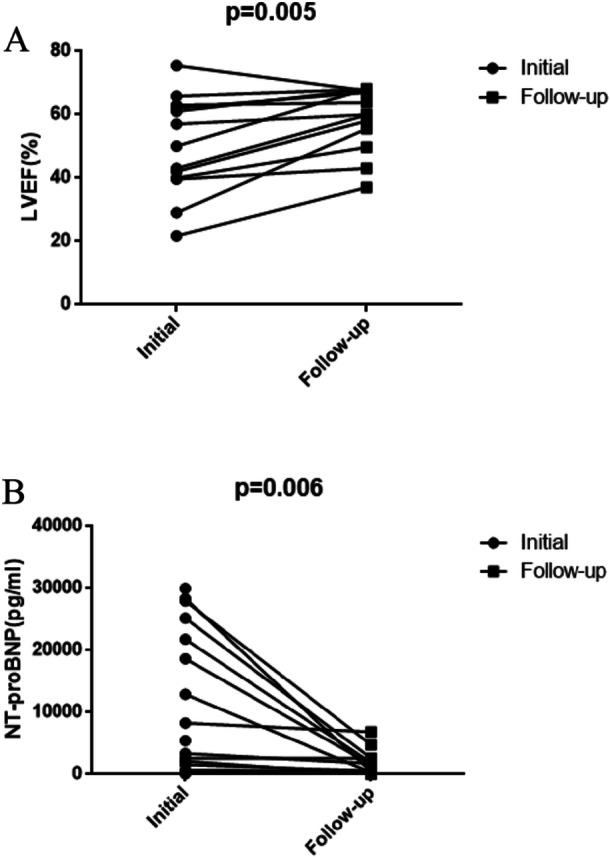
(A) Level of left ventricular ejection fraction (LVEF) at the initial stage and at the last visit. (B) Level of N‐terminal pro‐B‐type natriuretic peptide (NT‐proBNP) at the initial stage and at the last visit.

With a median follow‐up of 4 (range, 1–24) months, 20 patients achieved symptom remission. The NT‐proBNP level significantly declined from 11,838.9 ± 11,108.3 to 1,849.1 ± 2006.1 pg/mL (*p* = 0.006). A total of 13 patients underwent follow‐up echocardiography, which revealed a significant improvement in LVEF from 49.9 ± 15.7 to 58.9 ± 10.2 (*p* = 0.005) (Figure 1). Four patients were transferred to the intensive care unit for advanced life support, of whom 2 (9.1%) died due to heart failure. Three patients experienced an initial improvement, followed by a relapse.

The three patients who received RTX treatment initially experienced a rapidly progressive disease course. Cases 1 and 2 received methylprednisolone pulse therapy in addition to RTX, whereas Case 3 was administered high‐dose corticosteroids. All three patients showed clinical improvement within a mean of 2 weeks, with cardiac function recovering from NYHA Classes II–IV to Class I within 2 months. Initially, troponin‐T and NT‐proBNP levels significantly decreased after RTX treatment in Cases 1 and 2. Case 3 underwent lymphocyte subset testing before and after RTX administration (4 weeks after the second infusion). The results indicated a decline in CD4 + T cells (from 48.4% to 30.7%) and B cells (from 5.2% to 0%). Conversely, the CD8 + T cells increased from 41.0% to 63.7%, and the natural killer cells slightly increased from 4.5% to 5.5%. Follow‐up echocardiography was performed at months 1, 3, and 4. All the wall motion abnormalities completely resolved, returning to normal. In addition, LVEF returned to normal levels (Table 3). Meanwhile, the GCs were rapidly tapered to moderate‐to‐low doses (prednisone 0.3–0.5 mg/kg/d) within 1 month.

**Table 3:** Cardiac assessment before and after RTX treatment.

	Before RTX	After RTX
NYHA		
Case 1	IV	I
Case 2	IV	I
Case 3	II	I
Troponin‐T (normal 0–14 pg/mL)		
Case 1	63	23
Case 2	31	11
Case 3	3	4
NT‐proBNP (normal < 125 pg/mL)		
Case 1	18,655	1538
Case 2	35,000	150
Case 3	69	59
Hydropericardium		
Case 1	Yes	No
Case 2	Yes	Yes
Case 3	Yes	No
Wall motion abnormalities		
Case 1	Yes	No
Case 2	Yes	No
Case 3	Yes	No
LVEF (%)		
Case 1	40	53
Case 2	29	62
Case 3	53	58

Abbreviations: LVEF, left ventricular ejection fraction; NA, not available; NT‐proBNP, N‐terminal pro‐B‐type natriuretic peptide; NYHA, New York Heart Association; RTX, rituximab.

## 4 Discussion

Among the many phenotypes, LM tends to predict more severe disease activity and is one of the main causes of mortality in patients with SLE. This study demonstrated comparable mortality rates to previous studies [3, 14, 15], and cardiac symptoms in 50% of patients occurred prior to the diagnosis of SLE. Unlike other myocarditis etiologies, LM has a low incidence of chest pain and has nonspecific clinical manifestations. This highlights the importance of awareness of this rare cause of cardiac symptoms among rheumatologists and cardiologists.

Antibodies play a major pathogenic role in SLE development and progression. Previous studies have identified several antibodies potentially related to LM, although the results were inconsistent [3, 14, 15, 16, 17]. The present study showed that compared with patients with SLE without LM, those with LM had significantly higher rates of anti‐SSA and anti‐SSB antibody positivity, which impacts cardiac conduction tissue. Maternal anti‐SSA and anti‐SSB antibodies are known to cross the transplacental barrier *in utero*, accumulate in the fetal myocardium, and cause inflammation, fibrosis, and ultimately irreversible myocardial damage [18, 19]. A possible mechanism is that anti‐SSA and anti‐SSB autoantibodies can cross‐react with L‐ and T‐type calcium channels in the heart, impairing calcium currents and disrupting calcium homeostasis [20]. However, the role of these antibodies in the pathogenesis of myocardial injury in adult patients with SLE remains unclear. A recent study reported that the presence of anti‐SSA, in combination with anti‐SSB, may amplify the autoimmune inflammatory response, exacerbating cardiac structural damage [21]. Therefore, early cardiac evaluation is crucial in patients with SLE exhibiting anti‐SSA and/or anti‐SSB antibody positivity. In addition, closer cardiovascular monitoring is needed to prevent severe complications.

Unlike previous studies [14, 22], the present study found no statistically significant difference in SLEDAI 2 K score between patients with SLE with and without LM, even though all patients with SLE demonstrated moderate‐to‐severe disease activity. However, the SLE‐DAS was considerably higher in patients with LM (29.86 ± 7.88 vs. 15.79 ± 9.66). It is well recognized that cardiopulmonary assessment in the SLEDAI‐2K score is limited to pericarditis and pleurisy. Contrarily, SLE‐DAS includes assessment of additional organ involvement, such as interstitial pneumonia, diffuse alveolar hemorrhage, pulmonary hypertension, and myocarditis. Therefore, SLE‐DAS appears to be more suitable in cases of cardiopulmonary involvement, consistent with our previous study [23].

In our cohort, the prevalence of LN was much higher in patients with LM than in those with SLE without LM, which is consistent with a previous study [3]. LM is believed to be an immune‐complex–mediated disease that results in complement activation, inflammation, and myocardial injury. Given that cardiorenal pathophysiology is interrelated, Nicholas A Young et al. found that autoimmune myocarditis developed before glomerulonephritis in a murine model of LN. Histological analysis of the mice revealed myocarditis and fibrosis, whereas immunohistochemical staining revealed the presence of CD4+ and CD8 + T cells and IL‐17 in cardiac infiltrates [24]. Furthermore, T‐cell subset imbalance was involved in the pathogenesis of LM [25] and LN [26]. Patients with autoimmune myocarditis had reduced regulatory T cells, alongside elevated helper T (Th) 1 and Th17 cell populations [27]. In patients with LN, increased frequencies of Th17 and Th1 cells have been consistently observed [26]. However, it is noteworthy that in patients with LN who present with chest tightness and/or pain, differential diagnosis is crucial. In particular, prompt detection of heart failure, myocardial infarction, or myocarditis is essential.

Echocardiography is frequently employed in LM diagnosis. Compared with patients with other causes of myocarditis, the present study demonstrated that patients with LM were more likely to develop wall motion abnormalities, reduced LVEF, and valvular regurgitation. However, chest pain was less frequent in patients with LM. Notably, the clinical manifestations of LM are subtle and nonspecific. Elevated troponin‐T and NT‐proBNP levels may help increase the sensitivity of LM diagnosis.

To date, no randomized clinical trials have investigated the treatment effects on LM. Nevertheless, there have been reports of the effectiveness of traditional therapies, such as GCs, IVIG, MMF, and CTX, for LM [14, 15, 16], whereas the use of biologics for LM treatment has been rarely documented in the literature. In our cohort, three patients with LM were treated with RTX in addition to GCs, and all of them experienced rapid recovery. Furthermore, RTX exerts a favorable steroid‐sparing effect. A previous case report also described three patients with LM treated with RTX, and all of them recovered from myocardial dysfunction after the RTX therapy [28]. The primary therapeutic mechanisms of RTX are B‐cell depletion, and the effect of RTX on T‐cell subsets has been explored. RTX can effectively inhibit CD4 + T‐cell activation and partially restore the balance of T‐lymphocyte subsets [29]. In patients with SLE‐associated Evans' syndrome, the serum levels of tumor necrosis factor α were also significantly reduced following RTX treatment, whereas no apparent change in the serum levels of IL‐6, sIL‐2 receptor, and transforming growth factor β1 was observed [30]. However, in our study, the lymphocyte subsets before and after RTX infusion were available for only one patient, and cytokine data were completely lacking. Further studies are warranted to elucidate the effectiveness and mechanism of RTX, particularly in LM treatment. Although our data revealed no differences in HCQ intake between the groups, it is worth noting that the lack of HCQ use, the background therapy for SLE, was associated with increased risk of myocarditis [31]. However, the cardiotoxicity of HCQ cannot be overlooked. On light microscopy, chloroquine cardiomyopathy may present with diffuse myocardial fiber enlargement, fiber‐size variation, endocardial fibrosis, and myocyte vacuolization [32]. Biopsy remains the only definitive method for distinguishing HCQ cardiomyopathy from LM, which is undoubtedly a great challenge.

This study has limitations that need to be acknowledged. The diagnosis of LM was mainly based on clinical symptoms, serological tests, and images, and the absence of EMB was a major study limitation. As all patients were enrolled from a single center, a potential selection bias could not be excluded, and the small sample size constrains the statistical analysis. Furthermore, some patients did not undergo follow‐up echocardiography after discharge as their clinical symptoms had already improved. In addition, owing to the limited number of cases, the efficacy of RTX was not compared with that of traditional therapy, although the three patients who were treated with RTX yielded promising results. Finally, our data is insufficient to explore the pathophysiological mechanism of LM owing to the limitations of the retrospective study. Further studies are warranted to elucidate the pathophysiological mechanism of LM and to establish optimal therapeutic regimens for LM.

## 5 Conclusions

In summary, LM is a rare SLE complication associated with a high mortality rate. Compared with patients with SLE without LM, those with LM frequently developed LN, had a higher rate of anti‐SSB antibody positivity, and more often exhibited abnormal echocardiographic findings. RTX is a potentially promising option for LM treatment, though further studies are needed to confirm its efficacy. Interestingly, our study also revealed that SLE‐DAS may be a better tool for evaluating the disease activity of SLE than SLEDAI 2 K in patients with cardiac involvement.

## Author Contributions

Mengxue Yan and Siping Li acquired the data. Dong Yan and Siping Li performed the data analysis and drafted the manuscript. Zhichun Liu and Leixi Xue provided the conception, design, and critical revisions to the manuscript. All authors have read and approved the final manuscript.

## Conflicts of Interest

The authors declare no conflicts of interest.

## Data Availability

The data that support the findings of this study are available from the corresponding author, Leixi Xue, upon reasonable request.
